# Plasma Functional Proteins and Peptides: A Sustainable Nutritional Alternative to Support Piglet Performance and Health

**DOI:** 10.3390/ani16081256

**Published:** 2026-04-19

**Authors:** Javier Polo, Yanbin Shen, Joe Crenshaw, Núria Tous, David Torrallardona

**Affiliations:** 1 APC, LLC, Ankeny, IA 50021, USA; yanbin.shen@apcproteins.com (Y.S.); joe.crenshaw@apcproteins.com (J.C.); 2 APC Europe, S.L.U., 08403 Granollers, Spain; 3 Institut de Recerca i Tecnologia Agroalimentàries (IRTA), 43120 Constantí, Spain; nuria.tous@irta.cat (N.T.); david.torrallardona@irta.cat (D.T.)

**Keywords:** nursery diets, spray-dried plasma, hydrolyzed plasma, zinc oxide alternative, sustainable pig farming, circular economy, feed efficiency

## Abstract

This study evaluated the effects of including spray-dried porcine plasma (SDP) in nursery diets and enzymatically hydrolyzed plasma (EHP) in drinking water on piglet growth performance and post-weaning diarrhea (PWD). Four treatments were tested: CONTROL (soy protein concentrate, SPC), P1SDP (5% SDP in phase 1), P1 + P2SDP (5% SDP and 2% SDP in phases 1 and 2), and EHP (0.88% in water during phases 1 and 2). No significant differences among treatments were observed during phase 1. During phase 2 (14–28 days) pigs fed SDP or pigs provided EHP in water had higher average daily gain (ADG; *p* = 0.001) and feed conversion (GFR; *p* = 0.013) versus the other groups. Pigs fed SDP in the first two phases had an average d-42 body weight that was 1.54 kg heavier than controls. Post-weaning diarrhea was not observed at any time during the study. These results support the use of SDP and EHP as effective nutritional strategies to enhance the growth and resilience of pigs during the post-weaning period. Both ingredients contribute to sustainable pig production by improving efficiency and promoting circular economy practices through the valorization of animal by-products.

## Simple Summary

Weaning is a stressful time for piglets and often leads to reduced feed intake, slower growth, and digestive issues such as post-weaning diarrhea (PWD). Traditionally, high levels of zinc oxide have been used to control PWD, but due to regulatory restrictions, alternative strategies are needed. This study evaluated the use of two plasma-derived functional ingredients, spray-dried porcine plasma (SDP) included in feed, and enzymatically hydrolyzed plasma (EHP) administered through drinking water, during the nursery period. Our findings showed that piglets fed SDP in both phase 1 (0–14 days post-weaning) and phase 2 (14–28 days post-weaning) diets had better weight gain and feed efficiency compared to those fed a control diet based on soy protein concentrate. In addition, providing EHP through the water line helped improve piglet performance in the second phase, even after an initial drop in feed intake. Importantly, no signs of diarrhea were observed in any treatment group. These results suggest that SDP and EHP can be valuable nutritional tools to improve piglet growth and health after weaning, offering sustainable alternatives to help producers manage nursery pigs without relying on pharmaceutical zinc oxide.

## 1. Introduction

The European Union (EU) ban on antibiotic growth promoters and the therapeutic use of zinc oxide has markedly influenced pig production practices. The reduction in therapeutic zinc oxide levels has heightened the clinical risk of edema disease caused by *E. coli*, prompting renewed industry focus on these issues [1]. A survey indicated that over 50% of German swine farms had animals that tested positive for the verotoxin gene, indicating susceptibility to edema disease [2]. In Europe, post-weaning diarrhea (PWD) is a leading cause of economic loss, with mortality rates reaching up to 20–30% in severe cases. Post-weaning diarrhea is further exacerbated by the presence of *E. coli* strains that exhibit resistance to conventional treatments and antibiotics [3,4]. Post-weaning diarrhea, together with pathogenic *E. coli*, not only compromises piglet survival but also impedes growth and escalates medical treatment costs, directly affecting farm profitability.

The weaning process and the transition to solid feed are critical periods for piglets. Optimal early-stage nutrition is crucial for ensuring piglet health and well-being. During the initial three to five days post-weaning, it is common for piglets to exhibit minimal to no feed intake. This period of reduced intake deprives the intestinal lumen of essential nutrients, resulting in adverse physiological effects such as intestinal villus atrophy, diminished enzyme activity, impaired nutrient absorption, increased intestinal permeability, and inflammation [5]. Collectively, these conditions contribute to reduced body weight (BW) gain during this critical phase. Additionally, the presence of unabsorbed nutrients, particularly proteins, within the intestinal lumen can lead to undesirable fermentation processes, which are recognized as risk factors for digestive disorders and diseases [5]. It is well-established that achieving high feed intake shortly after weaning is associated with improved gastrointestinal development and enhanced performance throughout the nursery stage, regardless of initial BW at weaning [5,6].

Spray-dried plasma (SDP) is a safe and functional protein ingredient that offers a promising solution to weaning stress by supporting sustained growth and mitigating health challenges. SDP is rich in bioactive peptides, growth factors, amino acids, and vitamins, all of which retain their biological activity post-processing [7]. Spray-dried plasma is not only a functional and highly digestible protein that supports growth and helps reduce health challenges in piglets, but it also contributes to sustainable farming practices. As a by-product of the meat industry, SDP aligns with the principles of the circular economy by upcycling materials that would otherwise go to waste. This reduces the environmental footprint of livestock production, making SDP a key ingredient in balancing piglet performance and sustainability.

Traditionally, SDP has been commonly included in pig starter diets, calf milk replacers, and broiler feed [8,9,10] to support animal growth and health during periods of stress. The functional components of dietary SDP enhance intestinal integrity and modulate the immune system, thereby mitigating the adverse effects of inflammation and stress [7]. Moreover, dietary SDP has shown a systemic effect with beneficial effects on other mucosal tissues, including lung-associated lymphoid tissue [7] and has been associated with improved growth performance and reduced mortality, particularly under conditions of environmental or pathological stress [8,11]. Consequently, SDP is considered a potential alternative to antibiotics and therapeutic ZnO [8,12]. Furthermore, SDP is a highly digestible protein that can be incorporated into weaning diets with higher protein levels without increasing the risk of PWD, thus improving overall performance by the end of the nursery period. Pigs fed diets with SDP have consistently been shown to enhance feed intake during the early weaning period, thereby avoiding the intestinal issues associated with low intake during these critical days.

A commercial enzymatically hydrolyzed spray-dried plasma (EHP; Pepteiva^®^, APC Europe, S.L., Granollers, Spain) that contains 73% protein and 14.5% ash is a concentrated fraction of bioactive peptides naturally present in plasma. When used in its dry form in feed, EHP has been shown to enhance performance in pigs [13] as well as in other animal species [14]. Enzymatically hydrolyzed plasma is also well-suited for administration via drinking water due to its high solubility in water and stability at acidic pH, and it offers a practical and flexible application route in drinking water to supplement plasma-derived bioactive peptides during periods of stress.

The experimental objectives were to evaluate the effects on performance and diarrhea scores of pigs fed porcine SDP in phase 1 or phases 1 and 2 diets compared to pigs fed a control phases 1 and 2 diet with soy protein concentrate. In addition, EHP was administered in the drinking water during nursery phases 1 and 2 to a group of pigs that were fed the control diets to evaluate the effects on performance and diarrhea scores compared to the other dietary treatment groups. We hypothesized that the inclusion of SDP in phase 2 diets would enhance piglet growth performance during the nursery period, and that supplementation with bioactive peptides from enzymatically hydrolyzed plasma (EHP) via the drinking water could further support piglet development.

## 2. Materials and Methods

The animals in this experiment were raised and treated according to the Directive 2010/63/EU of 22 September 2010, and the recommendation of the European Commission 2007/526/CE covering the accommodation and care of animals used for experimental and other scientific purposes, and the Spanish Royal Decree 118/2021 principles for animal care and experimentation. This experiment was also approved by IRTA’s Ethical Committee for Animal Experimentation (CEEA).

The experiment was conducted at the IRTA research farm (Mas Bové, Tarragona, Spain) where pigs were housed in two identical weaning rooms with 16 pens each (1.84 m^2^) providing 0.46 m^2^ per pig. Each pen contained a single feeder with four eating spaces and one water drinking bowl to allow ad libitum feed and water consumption. The rooms were provided with automatic heating, forced ventilation, and completely slatted floors. The facilities were not cleaned and disinfected before the start of the trial to provide an unspecific stress challenge to the animals, aiming for “less experimental” conditions to simulate commercial environments.

The 26-day-old ([Large White × Landrace] × Pietrain) pigs were weaned, individually weighed, and randomly assigned by initial BW and pen location blocks to provide 8 pens/treatment with 4 pigs/pen (32 pigs/treatment; 128 total pigs) using a randomized complete block experimental design. Pigs were fed a three-phase nursery regimen formulated to normal protein levels (20.5%, 20.0% and 18.2%) per respective phases 1 to 3 diets using IRTA standard nutrient recommendations (Table 1). There were 14/day feeding durations for each dietary phase during the 42-day experiment, with phase 1 starting on day 1 postweaning. The experimental diets were mixed and prepared in pelleted form at the IRTA feed mill.

There were four treatment groups, including a CONTROL, P1SDP, P1 + P2SDP, and EHP group that were fed a three-phase nursery feed regimen. The CONTROL group was fed phases 1 to 3 control diets. The P1SDP group was fed the 5% SDP phase 1 diet and the control phases 2 and 3 diets. The P1 + P2SDP group was fed the 5% SDP phase 1 diet, the 2% SDP phase 2 diet, and the control phase 3 diet. The soy protein concentrate used in phases 1 and 2 diets was replaced by the levels of SDP used in phases 1 and 2 diets (Table 1). The EHP group was fed phases 1 to 3 control diets, while receiving 0.88% EHP supplementation via the drinking water during phases 1 and 2. Only pens assigned to the EHP treatment received the EHP solution in the water line. The EHP solution was continuously administered (24 h/d) in the drinking water using a waterline medicator (model D25RE5, Dosatron International S.A.S, Tresses, France) set to deliver a 4% solution. A fresh 22% stock solution of the EHP was prepared every three days and adjusted to pH 4.5 using citric acid. The water lines were flushed with a 0.1% solution of bleach, equivalent to 40 ppm active chlorine between EHP batches every three days to remove any potential microbial biofilm and to ensure hygiene and product stability throughout the supplementation period. Since this was the first experiment done to evaluate the application of EHP in nursery pig drinking water, procedures were developed before the experiment was started. The final titration of acidification of the EHP was determined to achieve a final pH of 4.5 to maintain 24 h microbial stability. The concentration of the stock solution (18%) was based on ease of mixability, water-line pressure, and medicator capacity to deliver the final solution of 0.88% EHP. All diets were iso-energetic, iso-protein and iso-SID lysine for all periods.

### 2.1. Performance and Feed Intake

Pigs were individually weighed on days 0, 14, 28 and 42 of the study and BW gain per phase or cumulatively over the study phases was used to calculate average daily gain (ADG). At the end of each feeding phase, the remaining feed in each hopper was recorded to calculate average daily feed intake (ADFI) and feed conversion ratio (GFR) per feeding phase. The remaining feed from the previous phase was fully removed at each phase change. Animals had ad libitum access to feed and water throughout the entire evaluation period. Every morning, all hoppers were checked visually and manually refilled as needed from individual feed bags prepared for each pen.

Although the pigs in the EHP group were fed control diets and received 0.88% EHP in drinking water during phases 1 and 2, the nutrient contributions from EHP were excluded from the ADFI results because water intake could not be measured. Therefore, ADFI and GFR results including phase 1 and 2 data for the EHP group should be interpreted with caution, considering that the total nutrient intake from feed and water was not included in the ADFI calculation.

### 2.2. Mortality and Morbidity

The general health status of the animals was monitored daily and registered throughout the trial. Mortality and morbidity were monitored daily, and animals exhibiting poor performance were excluded from the evaluation and counted as culled animals (including pig weight at the point of removal). Under veterinary supervision, the exclusion criteria requiring removal for individual care included signs of severe illness, extreme deviations in growth, or welfare concerns, referred to as pigs showing persistent anorexia, lethargy, and an inability to stand or compete for feed. The number and date of dead or culled piglets and their respective body weights (BW) were recorded to allow for corrections in ADFI, GFR, and ADG, as well as the reason for removal/death. In addition, the individual weight of the piglets remaining in the pen of the dead/culled animal was also recorded and used for the corrections.

### 2.3. Diarrhea Score

Every day of the study, the feces from each pen were visually examined in the morning to determine the incidence of post-weaning diarrhea and ascertain the health status of the pigs. The fecal evaluation used a score based on a five-point scale ranging from 0 to 4 (0 = firm and shaped; 1 = soft and shaped; 2 = soft without shape; 3 = loose; 4 = watery). Fecal scores 3 and 4 were considered as potential or confirmed diarrhea, respectively. However, there were no fecal scores = 4 recorded on any day of the study. Fecal scoring was always conducted by the same person. For statistical analysis, the highest fecal scores from each pen and period were used.

### 2.4. Statistical Analaysis

Normality of growth performance variables was confirmed using the Shapiro–Wilk test (*p* > 0.05) implemented in SAS (SAS Inst. Inc., Cary, NC, USA, version 9.4) via the PROC UNIVARIATE procedure and data were analyzed using the General Linear Model procedure of SAS (SAS Inst. Inc., Cary, NC, USA, version 9.4) with the pen as the experimental unit. Treatment and block were included in the model as main effects. All performance results are reported as least squares means of 8 pens per treatment (Table 2). Fecal consistency was analyzed using a Chi-square test of independence utilizing the PROC FREQ procedure of SAS (SAS Inst. Inc., Cary, NC, USA, version 9.4). Treatment effects with *p* < 0.05 were classed as significant or as trends if *p*-values ranged from 0.05 to 0.10. Tukey’s HSD post hoc test was used to determine differences among treatments for parameters having a significant treatment effect.

## 3. Results

The ADFI and GFR results including phase 1 and 2 data for the EHP group should be interpreted with caution considering that the total nutrient intake from feed and water was not included in the ADFI calculation.

During phase 1, there were no significant differences in performance or frequencies of fecal score among treatment groups (Table 2 and Table 3). Pigs in the EHP group tended (*p* = 0.055) to have reduced ADFI compared with pigs in the P1 + P2SDP and P1SDP groups.

During phase 2, pigs in the P1 + P2SDP and EHP groups had higher ADG compared to the CONTROL group and ADG was also higher for the EHP than the P1SDP group. A tendency (*p* = 0.096) for higher ADFI during this period was observed in the P1 + P2SDP group compared to the P1SDP group, and an improvement in GFR was observed in the EHP compared to the CONTROL group (*p* = 0.013). A tendency (*p* = 0.078) for higher d 28 BW in the P1 + P2SDP and EHP groups relative to the CONTROL group was observed.

During phase 3, when all groups were fed an identical phase 3 control diet, no significant differences among groups were observed for all performance parameters.

Considering cumulative results for phases 1 and 2 (d 0–28), pigs in the P1 + P2SDP tended (*p* = 0.082) to have higher ADG compared to the CONTROL group, but no differences in ADFI were observed. During this period, GFR was improved in the EHP group compared to all other groups (*p* = 0.001).

No significant differences among treatments were observed for cumulative performance parameters over the 42-day study. The BW on d 42 was 0.31 kg, 1.54 kg and 1.20 higher for P1 + P2SDP, P1SDP and EHP, respectively, compared to the CONTROL group.

There were no differences among dietary treatment groups for the fecal score frequencies (Table 3), and no incidence of diarrhea (fecal score = 4) was recorded on any day of the study. There were no individual pig or pen medical treatments given during the study. However, four piglets presented signs of meningitis and were immediately removed from the trial (two from treatment P1 + P2SDP during phase 2, one from treatment P1SDP during phase 3, and one from treatment EHP, also during phase 3). They were removed before medical treatment due to the rapid onset of meningitis symptoms. The data from these animals were not used for the calculations of pen performance. Their feed intake, for each feeding phase, was estimated from the feed intake of their pens until their removal, their weight gain, and the weight gain of their pen mates, according to Lindemann and Kim [15] and subtracted from the pen feed intake.

## 4. Discussion

Understanding and addressing the nutritional and health challenges during weaning is critical for pig producers to improve piglet outcomes and maintain farm profitability. In the current EU landscape, with a ban on antibiotic growth promoters and the therapeutic use of zinc oxide, it is common to see weaning diets with protein levels around 15–16% to avoid indigestible proteins entering the hindgut that can be used as substrate for the growth of pathogenic bacteria [16,17,18,19,20,21]. However, these lower protein levels can negatively impact animal performance during the nursery phase, leading to final weights that fall short of those expected at the end of this period [22].

The current study used normal protein levels during phases 1 and 2, along with the recommended SDP inclusion levels for these phases (5% and 2%, respectively). There were no statistically significant differences in performance among treatments during phase 1 (0–14 days). The lack of detection of significant differences among treatments in phase 1 may be due to the higher variability of the data associated with protocol requirements, as the facility and pens were not cleaned and sanitized before starting the experiment to exacerbate an unspecified weaning stress on the pigs. Under such study conditions, higher variability of performance data could be expected, and a higher number of replicates may have been needed to detect significant differences among treatments. Furthermore, PWD was not observed at any time during the study, and the apparent absence of an enteric pathogen challenge may have limited the magnitude of the pig performance response to SDP in phase 1.

During phase 2 (d 14–28), the P1 + P2SDP pigs fed 2% SDP had higher ADG and ADFI compared to the CONTROL group or the P1SDP group that received 5% SDP only during the first 14 days. By the end of the nursery phase, the final d-42 BW was 1.54 kg higher in the group fed SDP during phases 1 and 2 (P1 + P2SDP) compared to the CONTROL group, confirming results from Castelo et al. [23], who reported performance benefits and reduced *E. coli* K88 fecal shedding of non-restricted protein nursery diets with SDP included in phase 1 and 2 diets. In the study by Bailey et al. [24] using 2% SDP in a phase 2 diet with a normal crude protein level improved performance compared with the control group or the group receiving SDP only during phase 1. Their results demonstrated the benefits of supplementing SDP for longer periods, especially after transitioning from a phase 1 low protein diet to a phase 2 higher protein diet. In a related study [25], 5% spray-dried plasma (SDP) was included in a phase 1 diet (weeks 1 and 2) and compared to diets that continued SDP supplementation at decreasing levels of 5%, 2.5%, and 1% by phase during weeks 1–2, 3–4, and week 5, respectively. These treatments were evaluated against control diets without SDP, with or without pharmaceutical levels of zinc oxide. All diets were formulated to contain 17.1%, 17.4%, and 17.4% crude protein for phases 1, 2, and 3, respectively. The results showed that extending SDP inclusion into phases 2 and 3 produced performance outcomes (BW, ADG, and ADFI) comparable to those achieved with the zinc oxide control. Including SDP only during the first two weeks improved performance relative to the control without zinc oxide, though not to the same extent as the longer-duration SDP treatments. In contrast, when SDP levels were reduced by half, to either 2.5% in phase 1, or gradually decreased to 2.5%, 1.25%, and 0.5% across the three phases, no positive effects were observed. These findings indicate that both sufficient inclusion rates and extended supplementation are necessary for SDP to effectively support growth performance in the absence of therapeutic zinc oxide. Our results are consistent with these observations, emphasizing the importance of dietary level and feeding duration when using SDP in nursery pig diets.

In a recent study by Da Silva et al. [26] the impact of increasing cumulative SDP intake during the nursery phase on long-term pig performance, health, and carcass characteristics was evaluated. Although nursery growth performance was not significantly affected, pigs fed SDP diets exhibited reduced diarrhea severity and required fewer gastrointestinal medical treatments. Importantly, higher nursery SDP intake was associated with improved feed intake, weight gain, and final body weight during the grow-finish phase. Furthermore, all SDP diets fed during the nursery showed a significant reduction in lung lesions at slaughter compared to the control treatment.

Bailey et al. [24] reported that supplementing nursery diets with an adequate level of SDP during phase 1, followed by higher protein diets in phase 2 containing 2% SDP, supported piglet performance without increasing the risk of diarrhea. These benefits were likely related to the positive effects of SDP on intestinal barrier integrity, improved protein utilization, and reduced immune system activation, factors that are particularly important during the critical post-weaning period.

Despite having higher protein levels in the nursery diets, no PWD problems were observed during our study. This suggests that higher dietary protein levels are not necessarily correlated with increased diarrhea incidence, and that other factors, such as farm conditions, management practices, and overall pig health status, play critical roles in preventing PWD. Deng et al. [27] proved that the addition of very digestible animal protein like fish meal, poultry meal or SDP in phases 1, 2 and 3 replacing 0%, 33%, 66% or 100% of soy protein concentrate linearly increased final BW, ADG and ADFI without reporting diarrhea problems, similar to what we observed in our study. In addition, Bailey et al. [24] reported improved protein utilization, as indicated by lower plasma urea N, when pigs were fed normal CP phase 2 diets containing SDP. Therefore, SDP can be considered a key ingredient in nursery diets, effectively addressing the challenge of reducing dietary protein to prevent PWD, while simultaneously improving growth performance, even in the absence of therapeutic levels of zinc oxide.

The application of enzymatically hydrolyzed plasma in the water for pigs fed control diets (P1 + P2EHP) in our study suggests that after an initial adaptation period in phase 1, pigs supplemented with EHP via the water line demonstrated significantly improved ADG in phase 2. Delivering EHP through drinking water showed similar ADFI in phase 1 as the CONTROL group, but higher ADG and ADFI in phase 2 compared to the CONTROL and similar to the P1 + P2SDP group. These observations could be a result of a higher overall dry matter (protein and energy) intake during phases 1 and 2 because the pigs consumed both the standard balanced diet and additional peptides and nutrients provided via the water. Moreover, the use of citric acid to stabilize the EHP solution could have also contributed to the improved phase 2 growth performance because organic acids are known to support gut health and nutrient absorption. The EHP is rich in functional peptides derived from plasma, which have been previously shown to enhance growth in piglets when included in feed [13,14].

This is the first reporting EHP use in drinking water for nursery pigs. One of the primary motivations for testing EHP in the water line was to capitalize on its high solubility and ease of application. On commercial farms, changing the feed formulation in response to acute health or performance issues is often not feasible. However, water-based supplementation of EHP offers a practical and immediate alternative to support animals under stress. Although PWD did not occur in this study, the positive results on performance suggest that EHP supplementation via drinking water could serve as a valuable nutritional strategy to supplement routine nursery feed regimens, and also be used as nutrient supplementation or intervention in other periods of swine production when nutrient intake is suppressed due to stressful events.

There are some limitations that should be considered when interpreting the results. The study was conducted under specific farm conditions and management practices, which may limit the direct extrapolation of the findings to other production systems. In addition, only growth performance and fecal consistency were evaluated without any physiological or mechanistic parameters (e.g., intestinal microbiota, immune response, or gut morphology) being measured, which could have provided further insight into the modes of action of SDP and EHP. Furthermore, water intake was not measured during the study; therefore, the actual nutrient intake in the group receiving enzymatically hydrolyzed plasma via the drinking water could not be accurately quantified and should be interpreted with caution. Finally, the study was conducted under conditions with a low incidence of post-weaning diarrhea, which may have limited the ability to detect potential differences in fecal scores and health outcomes between treatments. Despite these limitations, the study provides relevant practical information on the use of dietary SDP and the application of EHP in drinking water under commercial-like conditions.

## 5. Conclusions

The results of this study demonstrate that incorporating 2% SDP in phase 2 diets significantly improved nursery performance, supporting greater growth rates and feed intake. Similarly, EHP delivered via drinking water proved to be an effective strategy for enhancing growth in phase 2 and may offer a practical nutritional solution when dietary changes are not feasible under field conditions.

As a by-product of the meat industry, plasma-derived proteins align with circular economy principles by upcycling animal resources that would otherwise go to waste. Overall, these findings reinforce the value of SDP and EHP as a balanced approach to providing reliable nutritional tools to manage post-weaning challenges and safeguard pig health, while maintaining optimal growth and supporting environmentally responsible farming practices.

## Figures and Tables

**Table 1:** Ingredient and nutrient composition of diets by nursery phase and feeding duration.

	Phase 1 (d0–14)		Phase 2 (d14–28)		Phase 3 (d28–42)
Diet by Phase ^1^	Control	5% SDP	Control	2% SDP	Control
Ingredients					
Barley	30.71	33.64	37.79	38.94	27.82
Maize	20.00	20.00	20.00	20.00	21.39
Wheat	-	-	-	-	20.00
SBM (48%CP) ^2^	22.58	22.24	25.95	25.84	23.34
Sweet milk whey	6.50	6.50	3.00	3.00	-
SPC ^3^	6.67	-	2.67	-	-
SDP ^4^	-	5.00	-	2.00	-
Dextrose	6.50	6.50	3.00	3.00	-
Animal fat	3.63	3.20	3.96	3.80	3.60
L-Lysine-HCL	0.35	0.23	0.39	0.34	0.53
L-Threonine	0.17	0.08	0.18	0.14	0.23
DL-Methionine	0.19	0.13	0.18	0.15	0.19
L-Tryptophan	0.04	0.01	0.04	0.03	0.05
L-Valine	-	-	0.01	-	0.08
Salt	0.52	0.25	0.58	0.47	0.48
Calcium carbonate	0.03	-	0.01	-	0.24
Dicalcium phosphate	1.68	1.80	1.82	1.87	1.61
Noxyfeed ^5^	0.02	0.02	0.02	0.02	0.02
Vit-Min complex ^6^	0.40	0.40	0.40	0.40	0.40
Calculated nutrients					
Crude protein, %	20.5	20.5	20.0	20.0	18.2
ME, MJ/kg	13.8	13.8	13.7	13.7	13.7
SID Lysine, %	1.28	1.28	1.25	1.25	1.20
SID Met + Cys, %	0.75	0.76	0.74	0.74	0.71
SID Thr, %	0.83	0.83	0.81	0.81	0.78
SID Trp, %	0.26	0.26	0.25	0.25	0.24
SID Ile, %	0.78	0.75	0.75	0.74	0.65
SID Val, %	0.87	0.95	0.85	0.87	0.81
Crude fat, %	5.36	5.04	5.84	5.71	5.63
Crude fiber, %	3.09	2.93	3.39	3.33	3.14
Calcium, %	0.75	0.75	0.75	0.75	0.75
Phosphorous, %	0.69	0.73	0.70	0.72	0.64

^1^ Treatment group CONTROL was fed control phase 1, 2 and 3 diets; P1SDP was fed the 5% SDP phase 1 diet and phases 2 and 3 control diets; P1 + P2SDP was fed the 5% SDP phase 1 diet, the 2% SDP phase 2 diet and the phase 3 control diet; EHP was fed phases 1, 2 and 3 control diets while receiving a 0.88% solution of EHP (Pepteiva^®^, APC Europe, S.L., Granollers, Spain) in drinking water during phases 1 and 2. ^2^ Soybean meal 48% crude proein. ^3^ Soy protein concentrate X-soy200 from CJ Bio, Seoul, Republic of Korea. ^4^ Spray-dried porcine plasma Appetein GS from APC Europe, S.L., Spain. ^5^ ITPSA, Barcelona, Spain. Contains BHT+ propyl galate (56%) and citric acid (14%). ^6^ The vitamin-micromineral premix provided the following quantities of vitamins and microminerals per kg of complete diet: vitamin A (retinyl acetate) 10,000 UI; vitamin D_3_ (Cholecalciferol) 2000 UI; vitamin E (all-rac-alpha-tocopheryl acetate) 25 mg; vitamin K_3_ (menadione sodium bisulfite) 1.5 mg; vitamin B_1_ (thiamine mononitrate) 1.5 mg; vitamin B_2_ (Riboflavin) 3.5 mg; vitamin B_6_ (pyridoxine hydrochloride) 2.4 mg; vitamin B_12_ 20 μg; nicotinic acid (niacin) 20 mg; calcium D-pantothenate 14 mg; folic acid 0.5 mg; biotin 50 μg; Fe (from FeSO_4_·H_2_O) 120 mg; I (from KI) 0.75 mg; Cu (from CuSO_4_·5H_2_O) 6 mg; Mn (from MnO) 60 mg; Zn (from ZnO) 65 mg; Se (E 8) (from Na_2_SeO_3_) 0.37 mg. SBM = Soybean meal.

**Table 2:** Performance parameters by nursery phase of pigs fed diets with SDP in phase 1 or SDP in phase 1 and 2 diets.

Treatment Groups	CONTROL	P1SDP	P1 + P2SDP	EHP	Pooled SEM	*p* Value
Spray-dried plasma, %	-/-	5.0/-	5.0/2.0	-/-		
EHP in water, % ^1^	-/-	-/-	-/-	0.88/0.88		
Phase 1 (d 0 to 14)						
Initial BW, kg	7.33	7.33	7.34	7.32	0.016	0.337
ADG, g/d	123	155	158	126	70.93	0.214
ADFI, g/d ^2^	190	221	232	179	40.80	0.055
GFR	0.64	0.69	0.68	0.69	0.089	0.538
d 14 BW, kg	9.05	9.50	9.55	9.09	0.569	0.198
Phase 2 (d 14 to 28)						
ADG, g/d	369 ^c^	373 ^bc^	417 ^ab^	431 ^a^	31.99	0.001
ADFI, g/d	526	517	576	556	49.30	0.096
GFR	0.71 ^b^	0.72 ^ab^	0.73 ^ab^	0.78 ^a^	0.039	0.013
d 28 BW, kg	14.22	14.72	15.39	15.13	0.888	0.078
Phase 3 (d 28 to 42)						
ADG, g/d	716	702	743	737	64.30	0.566
ADFI, g/d	855	879	892	895	68.20	0.639
GFR	0.84	0.80	0.84	0.83	0.068	0.759
d 42 BW, kg	24.25	24.56	25.79	25.45	1.474	0.153
Phases 1 + 2 (d0 to 28)						
ADG, g/d	246	264	287	279	31.9	0.082
ADFI, g/d	358	369	404	368	43.0	0.190
GFR	0.69 ^b^	0.72 ^b^	0.71 ^b^	0.76 ^a^	0.028	0.001
Overall (d 0 to 42)						
ADG, g/d	403	410	439	432	35.30	0.159
ADFI, g/d	524	539	567	543	42.80	0.281
GFR	0.77	0.76	0.77	0.80	0.038	0.323

^1^ Enzymatically hydrolyzed plasma was added in the drink water. ^2^ For the EHP group, nutrient contributions from water intake of the EHP were not included in ADFI calculations. ^a≠b≠c^ Parameter least squares treatment means of 8 pens per treatment with uncommon superscripts differ among treatments (*p* < 0.05).

**Table 3:** Frequencies of fecal scores by nursery phase of pigs fed diets with SDP in phase 1 or SDP in phase 1 and 2 diets.

Treatment Groups	CONTROL	P1SDP	P1 + P2SDP	EHP	Chi-Square *p*-Value
Spray-dried plasma, %	-/-	5.0/-	5.0/2.0	-/-	
EHP in water, % ^1^	-/-	-/-	-/-	0.88/0.88	
Phase 1 (d 0 to 14)					
Score 0	2/8	1/8	3/8	2/8	0.729
Score 1	3/8	3/8	1/8	1/8	
Score 2	2/8	2/8	3/8	1/8	
Score 3	1/8	2/8	1/8	4/8	
Score 4	0/8	0/8	0/8	0/8	
Phase 2 (d 14 to 28)					
Score 0	1/8	4/8	1/8	3/8	0.512
Score 1	1/8	0/8	2/8	1/8	
Score 2	6/8	4/8	5/8	4/8	
Score 3	0/8	0/8	0/8	0/8	
Score 4	0/8	0/8	0/8	0/8	
Phase 3 (d 28 to 42)					
Score 0	6/8	8/8	7/8	5/8	0.526
Score 1	2/8	0/8	1/8	3/8	
Score 2	0/8	0/8	0/8	0/8	
Score 3	0/8	0/8	0/8	0/8	
Score 4	0/8	0/8	0/8	0/8	

Fecal score was assessed for each pen (*n* = 8 pens/treatment) using a subjective score on a five-point scale ranging from 0 to 4 (0 = firm and shaped; 1 = soft and shaped; 2 = soft without shape; 3 = loose; 4 = watery). The highest score observed for each pen in each period was used for the calculation of frequencies. ^1^ Enzymatically hydrolyzed plasma was added in the drink water.

## Data Availability

All data from this study are provided in the manuscript. The raw data supporting the conclusions of this article will be made available by the authors upon request.
